# Radiological Changes in Chest Computed Tomography Findings of School Cooks: A Comparative Study With Age-matched Controls

**DOI:** 10.1016/j.shaw.2025.01.006

**Published:** 2025-01-30

**Authors:** Jung Hee Hong, Jin Young Kim, Kiook Baek

**Affiliations:** 1 Department of Radiology, College of Medicine, Keimyung University, Daegu, Republic of Korea; 2 Department of Occupational and Environmental Medicine, Dongguk University Gyeongju Hospital, Gyeongju, Republic of Korea; 3 Department of Preventive Medicine, College of Medicine, Dongguk University, Gyeongju, Republic of Korea

**Keywords:** School cooks, Computed tomography, Cooking oil fumes, Nodule

## Abstract

**Background:**

This study investigates subclinical radiologic changes in the respiratory system in school cooks with long-term exposure to cooking oil fumes.

**Methods:**

Using low-dose chest computed tomography screening data, we compared 88 school cooks to an age- and sex-matched control group of 88 individuals to assess the presence of lung nodules, bronchial wall thickening, and lymph node size variations.

**Results:**

The results demonstrated a significantly higher prevalence of solid nodules in cooks (75.0% vs. 33.0%, *p* < 0.001). Additionally, the thickness of the right bronchial wall (1.41 ± 0.19 mm vs. 1.33 ± 0.18 mm, *p* = 0.004) and the left bronchial wall (1.31 ± 0.21 mm vs. 1.25 ± 0.20 mm, *p* = 0.044) were significantly greater in the exposed group. Cooks also had larger lymph nodes at various sites: right paratracheal or hilar (4.12 ± 1.66 mm vs. 3.13 ± 1.36 mm, *p* < 0.001), left paratracheal or hilar (2.63 ± 1.50 mm vs. 1.98 ± 1.27 mm, *p* = 0.002), subcarinal (3.44 ± 1.58 mm vs. 2.47 ± 1.16 mm, *p* < 0.001), right inferior interlobar (3.90 ± 1.16 mm vs. 2.85 ± 0.90 mm, *p* < 0.001), and left inferior interlobar (3.83 ± 1.22 mm vs. 3.02 ± 1.11 mm, *p* < 0.001).

**Conclusion:**

This study demonstrated that individuals with long-term employment as school cooks exhibited subclinical yet detectable radiologic findings in the lungs.

## 1 Introduction

Numerous studies have reported that individuals working in food preparation facilities are exposed to various harmful substances during cooking processes, which may lead to various respiratory diseases, including lung cancer. Cooking oil fumes (COFs) are known to be produced frequently in Asian countries where high-temperature stir-frying with oil is common, and their health effects have been highlighted in previous studies [1]. COFs are a major source of indoor air pollution, containing a variety of toxic substances, including heterocyclic amines, polycyclic aromatic hydrocarbons (PAHs), and volatile organic compounds. In South Korean school cafeterias, short-term exposure to high concentrations of COFs containing CO, CO₂, and fine pareticulate matter (PM_2.5_) has been documented [2].

Occupational exposure to harmful substances generated during cooking, including COFs, is a significant concern not only in household environments but also in restaurants and large-scale food service facilities. Such exposure has been associated with multiple toxic effects on the respiratory system, likely through mechanisms involving genotoxicity and inflammation [3]. Furthermore, COFs have been classified as carcinogenic due to their toxic and epidemiologically significant effects [4,5]. Due to findings from various epidemiological and mechanistic studies, high-temperature cooking has been classified as “probably carcinogenic to humans” (Group 2A) by the International Agency for Research on Cancer [6]. Subsequently, a meta-analysis of studies investigating the association between COFs and lung cancer incidence reported that exposure to oil fumes during cooking significantly increases the risk of lung cancer, particularly among nonsmoking women [7]. However, given the influence of cooking styles, culinary culture, and cooking environments, applying toxicity and epidemiological findings on COFs from one cultural setting to another is challenging.

**Fig. 1:**
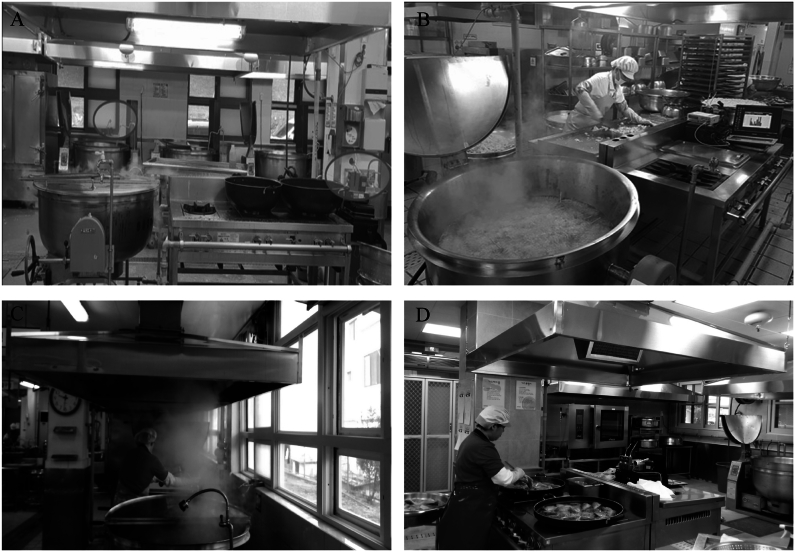
Overview of a Korean cafeteria kitchen. (A) A pot boiling soup on a gas stove and a large pan used for stir-frying. (B) A cooking staff member preparing ingredients for dishes. (C) A cooking staff member operating a large cooking pot. (D) A cooking staff member using a grill for oil-based frying. Reproduced with permission from Republic of Korea Occupational Safety and Health Research Institute, Airborne Hazardous Substances Generated During Cooking and Their Respiratory Health Effects (in Korean). Copyright © Republic of Korea Occupational Safety and Health Research Institute.

In Korean schools, an average of 437 students are served per school, with one main cook and 4.8 assistant cooks preparing meals [8]. Compared to Chinese cuisine, less oil is used in meal preparation. Typically, the meals include steamed rice, one soup, and 2–3 side dishes prepared by stir-frying, seasoning, or steaming. Cooking is primarily done using gas, although the number of schools utilizing electric cooking equipment is gradually increasing [2]. Each school employs a dedicated nutrition teacher responsible for professionally managing meal menus, ingredients, and recipes, creating a relatively standardized cooking environment across schools. An approximate cooking scene in Korean school is illustrated in Fig. 1 [9].

While studies on industrial hygiene exposure to COFs in the Republic of Korea have been conducted [2,10], there is limited research on the associated health effects. Identifying a significant increase in cancer risk requires large-scale, long-term cohort data. Nonetheless, even in relatively smaller datasets, several studies on respiratory hazards have demonstrated the feasibility of using computed tomography (CT) to detect subclinical respiratory changes, a widely adopted research method for investigating other hazardous agents [11,12]. Thus, this study aimed to examine subclinical respiratory changes in school cooks by comparing their chest CT scans to those of a nonexposed control group and to characterize these changes.

## 2 Materials and methods

### 2.1 Ethical statements

This retrospective study was approved by the institutional review board of Keimyung University Hospital (IRB number: DSMC 2024-07-002); the requirement for patients' informed consent was waived.

### 2.2 Study design and population

A retrospective review was conducted on school cooks who underwent low-dose chest CT screening at a single tertiary hospital from January 2022 to December 2022. The chest CT screening for cooks included participants aged 55 years or older or those who had been employed for over 10 years. To focus on long-term exposure to COFs, this study was limited to participants aged 45 to 54 years; thus, the group of school cooks comprised individuals who have been engaged in cooking duties in school cafeterias for at least 10 years. A total of 93 consecutive participants were identified during the study period. Among them, we excluded the participants with 1) a history of old tuberculosis (*n* = 3), based on CT findings showing evidence of old tuberculosis, 2) the presence of active pneumonia (*n* = 1), identified through CT imaging indicating active lung infection, and 3) a history of malignancy (*n* = 1), excluded based on CT scans taken at the same medical institution in the past, which had previously diagnosed malignancy. Finally, 88 cases were enrolled.

The control group consisted of individuals who visited a tertiary hospital's health promotion center and underwent low-dose chest CT scans for health screening, without the use of health insurance. In the Korean healthcare system, CT scans ordered for specific disease workups are eligible for national insurance claims; therefore, those in this group were considered free of any known active respiratory diseases. To ensure comparability, the control group was randomly selected and matched to the school cooks on a 1:1 basis, with each control having exactly the same age and sex as their paired cooks. The matching process was conducted blindly and randomly by a research assistant, who had access only to the age (years) and sex information of the participants and did not have permission to view the CT scans. This process ensured an unbiased selection of controls and resulted in 88 matched controls.

### 2.3 CT image analysis

**Fig. 2:**
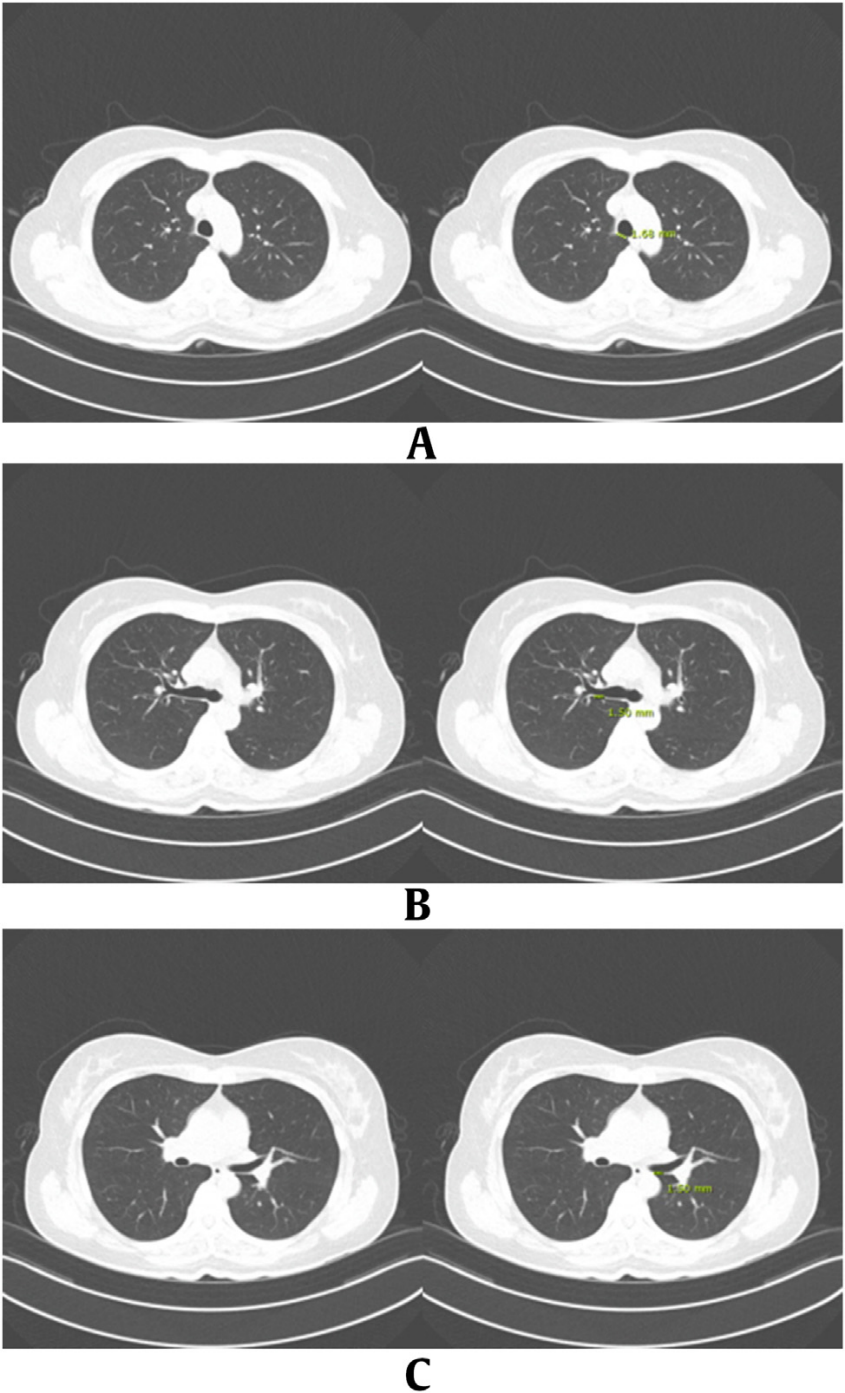
Methods of measuring bronchial wall thickness on CT image. Bronchial wall thickening was measured at three levels with most distinct location with minimal motion artifacts: (A) the trachea, just above to the azygos vein, (B) the right upper lobar bronchus, about 2–3 cm distal from the tracheal bifurcation, and (C) the left main bronchus, at the area positioned between the esophagus and the left lower lobar pulmonary artery, ensuring that no adjacent soft tissue structures interfered with the assessment of wall thickening. CT, computed tomography.

**Fig. 3:**
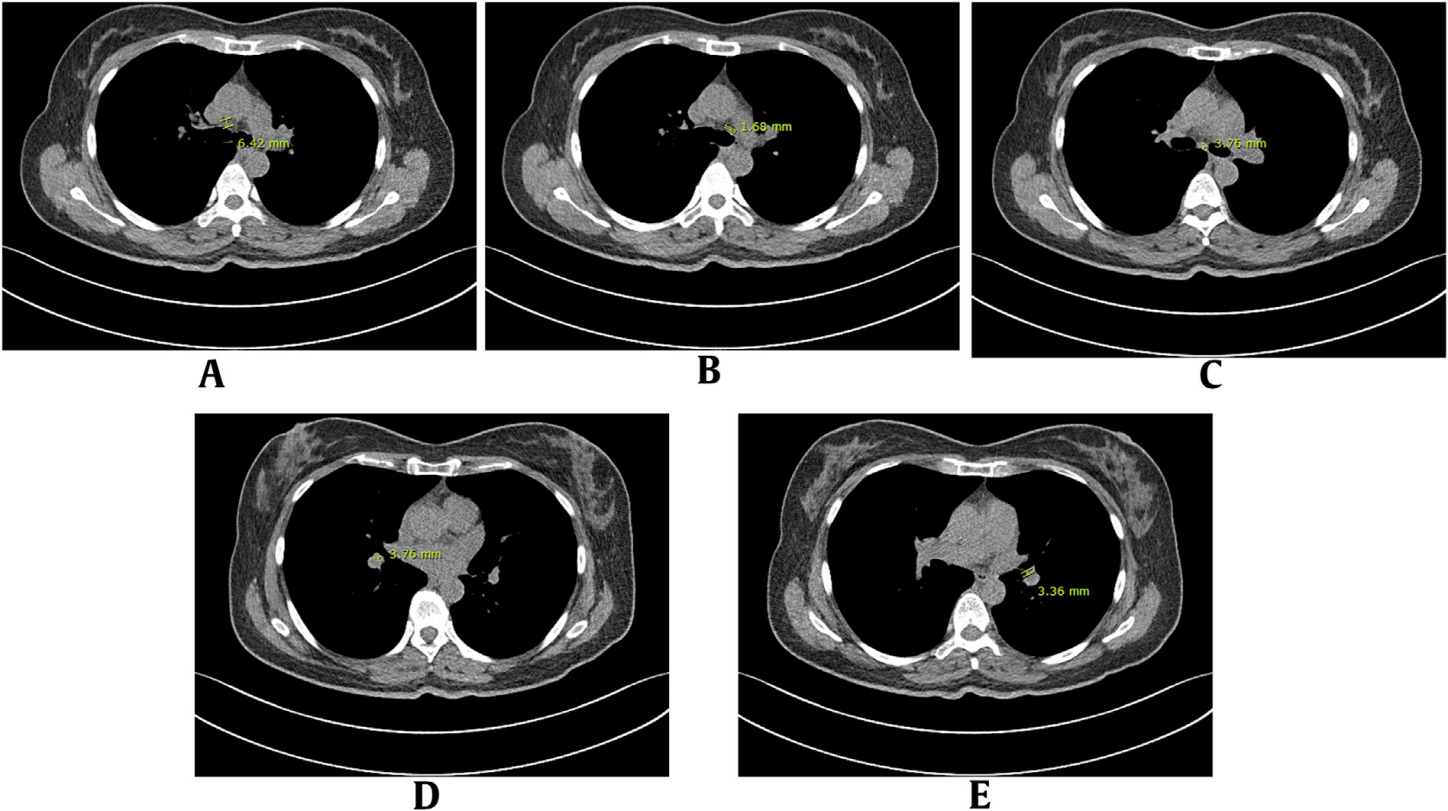
Methods of measuring short axis diameter of lymph nodes on CT image. (A) The right lower paratracheal or right hilar, (B) left lower paratracheal or left hilar, (C) subcarinal, (D) right inferior, and (E) left inferior interlobar lymph nodes were measured at the largest, most distinct location with minimal motion artifacts. CT, computed tomography.

Chest CT scans were evaluated by a thoracic radiologist (Jung Hee Hong, with 11 years of experience in chest imaging), who was blinded to the case–control classification. The assessments, including measurements, were subsequently verified by a second thoracic radiologist (Jin Young Kim, with 13 years of experience in chest imaging), who was also blinded to the classification. If the second radiologist deemed a revision of the measurements necessary, adjustments were made through a consensus reached between the radiologists. The nodule density was classified into three categories: pure ground-glass nodules (GGNs), part-solid nodules (PSNs), and solid nodules. The number of lung nodules was checked according to the nodule density (GGN, PSN, or solid) and categorized as follows: no nodules, group 1 (1–3 nodules), group 2 (4–6 nodules), group 3 (7–10 nodules), and group 4 (more than 10 nodules). Additionally, nodules were classified according to Lung Imaging Reporting and Data System (Lung-RADS) v2022, with category 1 (negative) and category 2 (benign appearance) designated as negative results and category 3 (probably benign) and category 4 (suspicious) designated as positive results [13,14]. The longest axis diameter of the largest nodule was measured and recorded according to the nodule density. Degrees of emphysema was graded on a 5-point scale (0, none; 1, trace or mild; 2, moderate; 3, confluent; and 4, advanced destructive) [15]. Presence of interstitial lung abnormality (ILA) was recorded as either absent or present [16]. We defined small airway wall thickening as a lumen occupying less than 80% of the total airway diameter, and this was scored as group 0 (absent), group 1 (1 segment involved), group 2 (2 segments involved), or group 3 (more than 3 segments involved) [17]. Bronchial wall thickening was measured at three levels at the most distinct location with minimal motion artifacts as possible: 1) the trachea, just above to the azygos vein, 2) the right upper lobar bronchus, about 2–3 cm distal from the tracheal bifurcation, and 3) the left main bronchus, at the area positioned between the esophagus and the left lower lobar pulmonary artery, ensuring that no adjacent soft tissue structures interfered with the assessment of wall thickening (Fig. 2) [18]. The short-axis diameters of 1) the right lower paratracheal or right hilar, 2) left lower paratracheal or left hilar, 3) subcarinal, 4) right inferior, and 5) left inferior interlobar lymph nodes were measured at the largest, most distinct location with minimal motion artifacts (Fig. 3).

### 2.4 Chest CT acquisition

All chest CT were performed using 64- or 128-slice multidetector CT scanners (SOMATOM Force, SOMATOM definition Edge, SOMATOM drive; Siemens Medical Solutions, Forchheim, Germany). The parameters for the CT were as follows: tube voltage, 120 kVp; tube current, reference: 20 mAs, with automatic exposure control; slice thickness: 1.0 mm and 3.0 mm; and filter: b49f. CT images were obtained in the supine position with craniocaudal direction during a single full-inspiratory breath-hold without contrast enhancement.

### 2.5 Statistical analysis

We used the chi-square test to compare categorical variables and the student *t* test to compare continuous variables. A *p* value less than 0.05 was considered to indicate statistical significance. A nonparametric approach was used when the sample size was below 30. All analyses were conducted using R project (https://r-project.org).

## 3 Results

### 3.1 General characteristics

All 176 participants (88 school cooks and 88 nonexposed controls) were female, with a mean age of 51.41 ± 2.42 years. We assessed differences in radiological findings between the two groups, focusing on solid nodules, GGNs, PSNs, bronchial wall thickness, and lymph node size, among other features (Table 1).

**Table 1:** Clinical and radiologic characteristics of 176 participants (88 school cooks and 88 control group)

	Degree	School cooks	Control group	*p*
Number		88 (100.0)	88 (100.0)	
Age (years)∗		51.41 ± 2.42	51.41 ± 2.42	>0.999
Emphysema†	Group 0	73 (83.0)	80 (90.9)	0.180
	Group 1	15 (17.0)	8 (9.1)	
	Group 2	0 (0.0)	0 (0.0)	
	Group 3	0 (0.0)	0 (0.0)	
	Group 4	0 (0.0)	0 (0.0)	
Presence of solid nodule†	Yes	66 (75.0)	29 (33.0)	<0.001‡
	No	22 (25.0)	59 (67.0)	
Solid size (mm)∗		3.18 ± 1.52	3.59 ± 1.68	0.271
Solid count†	No	22 (25.0)	59 (67.0)	<0.001
	Group 1	41 (46.6)	26 (29.5)	
	Group 2	18 (20.5)	0 (0.0)	
	Group 3	3 (3.4)	2 (2.3)	
	Group 4	4 (4.5)	1 (1.1)	
Presence of GGN†	Yes	8 (9.1)	2 (2.3)	0.104
	No	80 (90.9)	86 (97.7)	
GGN size (mm)∗	Yes	6.25 ± 2.05	7.5 ± 3.54	0.588
	No	80 (90.9)	86 (97.7)	
GGN count†	Group 1	8 (9.1)	2 (2.3)	0.104
	Group 2	0 (0.0)	0 (0.0)	
	Group 3	0 (0.0)	0 (0.0)	
	Group 4	0 (0.0)	0 (0.0)	
Presence of PSN†	Yes	3 (3.4)	0 (0.0)	0.246
	No	85 (96.6)	100 (100.0)	
PSN size (mm)∗		7.67 ± 0.58	—	—
	No	85 (96.6)	100 (100.0)	0.246
PSN count†	Group 1	3 (3.4)	0 (0.0)	
	Group 2	0 (0.0)	0 (0.0)	
	Group 3	0 (0.0)	0 (0.0)	
	Group 4	0 (0.0)	0 (0.0)	
Interstitial lung abnormality†	Yes	0 (0.0)	2 (2.3)	0.497
	No	88 (100)	86 (97.7)	
Small airway thickening†	Group 0	82 (94.3)	84 (95.5)	0.928
	Group 1	2 (2.3)	1 (1.1)	
	Group 2	1 (1.1)	1 (1.1)	
	Group 3	2 (2.3)	2 (2.3)	
	Group 4	0 (0.0)	0 (0.0)	
Tracheal wall thickening (mm)∗		1.43 ± 0.32	1.40 ± 0.22	0.465
Right bronchial wall thickening (mm)∗		1.41 ± 0.19	1.33 ± 0.18	0.004‡
Left bronchial wall thickening (mm)∗		1.31 ± 0.21	1.25 ± 0.20	0.044‡
4R/10R lymph node (mm)∗		4.12 ± 1.66	3.13 ± 1.36	<0.001‡
4L/10L lymph node (mm)∗		2.63 ± 1.50	1.98 ± 1.27	0.002‡
7 lymph nodes (mm)∗		3.44 ± 1.58	2.47 ± 1.16	<0.001‡
11R lymph node (mm)∗		3.90 ± 1.16	2.85 ± 0.90	<0.001‡
11L lymph node (mm)∗		3.83 ± 1.22	3.02 ± 1.11	<0.001‡

GGN, ground-glass nodule; PSN, part-solid nodule.

∗ Data are mean ± standard deviation.

† Data are number of patients, with percentages in parentheses.

‡ Indicates a significant finding.

### 3.2 Solid nodules, GGNs, and PSNs

The presence of solid nodules was significantly higher in the cook group than in the control group (66, 75.0% vs. 29, 33.0%; *p* < 0.001). However, when comparing the size and number of solid nodules between the two groups, no statistically significant differences were found. In terms of GGNs and PSNs, both nodule types were found to be less prevalent in the control group than in the cooks. Despite this, the absolute numbers of GGNs and PSNs were too small to perform meaningful statistical analyses. The small sample sizes for these types of nodules make it difficult to draw definitive conclusions about their significance in the two groups. In the context of Lung-RADS v2022, cooks had category 2 (*n* = 58), category 3 (*n* = 8), and category 4A (*n* = 2). In the control group, category 2 (*n* = 29), category 3 (*n* = 1), and category 4A (*n* = 1). Thus, the rate of positive results among cooks was 11.4% (*n* = 10; category 3, n = 8; category 4A, n = 2), whereas the control group showed a positive rate of 2.3% (*n* = 2; category 3, n = 1; category 4A, n = 1).

### 3.3 Bronchial wall thickness

When assessing bronchial wall thickness, the results showed no significant difference in tracheal wall thickness between the two groups. However, the thickness of both the right and left bronchial walls was significantly greater in school cooks than in controls (right bronchial wall thickening: 1.41 ± 0.19 mm vs. 1.33 ± 0.18 mm, *p* = 0.004; left bronchial wall thickening: 1.31 ± 0.21 mm vs. 1.25 ± 0.20 mm, *p* = 0.044). On the other hand, no significant difference was observed in small airway thickening between the two groups (5, 5.7% vs. 4, 4.5%, *p* = 0.928).

### 3.4 Lymph node size

The short-axis diameter of lymph nodes, including the right and left paratracheal or hilar (right, 4.12 ± 1.66 mm vs. 3.13 ± 1.36 mm, *p* < 0.001; left, 2.63 ± 1.500 mm vs. 1.98 ± 1.266 mm, *p* = 0.002), subcarinal (3.44 ± 1.58 mm vs. 2.47 ± 1.16 mm, *p* < 0.001), and interlobar lymph nodes (right, 3.90 ± 1.16 mm vs. 2.85 ± 0.90 mm, *p* < 0.001; left, 3.83 ± 1.22 mm vs. 3.02 ± 1.11 mm, *p* < 0.001), was significantly larger in school cooks than in controls.

### 3.5 Interstitial Lung Abnormalities (ILA)

Two cases of ILA were detected in the control group, while no cases were found in the school cook group (*p* = 0.497).

## 4 Discussion

In this study, we investigated low-dose chest CT in school cooks for over 10 years and compared them to a control group. The results demonstrated a significantly higher prevalence of solid nodules, bronchial wall thickening, and larger lymph node size in the school cook group than in controls. According to Lung-RADS v2022 criteria, the school cooks showed a positive rate of 11.4%, compared to 2.3% in the control group. No significant differences were observed in small airway thickening or ILAs.

COFs contain harmful compounds such as benzene, formaldehyde, 1,3-butadiene, aromatic amines, PAHs, and acrolein [[19], [20], [21], [22]]. These substances are known to trigger mutagenic and genotoxic effects, as well as cause inflammation or irritation in the airways. Recent researches indicate that COF-induced inflammation in the respiratory tract may lead to impaired lung function and be associated with chronic bronchitis, acute respiratory infection in children, chronic obstructive pulmonary disease, and lung cancer [21,23,24].

The composition and exposure levels of COFs vary depending on cooking styles and ingredients used. Notably, exposure to PAH-containing COFs is higher in regions with a culinary focus on oil-based stir-frying, as observed in several Asian countries [4]. Although the types of foods prepared can result in varying exposure levels in restaurants, school cafeterias, typically operated under local government budgets, follow standardized menus overseen by resident nutrition teachers, resulting in relatively similar meal preparations across schools in the Republic of Korea [25].

In the Republic of Korea, the median concentration of respirable dust measured in 55 school cafeterias was 38.37 μg/m³ [10]. Similarly, other studies conducted in Korean cooking facilities have reported that during oil-based cooking, CO concentrations can rise up to 295 ppm. The median level of PM_2.5_ was 24.78 μg/m³, with maximum concentrations reaching 367.90 μg/m³, demonstrating considerable variability depending on the cooking environment and methods used [2].

In animal studies using rats, COF exposure has been reported to cause acute airway injury in a dose–response relationship, associated with increased respiratory resistance due to airway secretion and decreased lung compliance in lung tissue [26]. Exposure to pollutants generated during cooking has been shown to induce health effects other than lung cancer even in young, healthy individuals. Studies on Chinese military cooks have reported a positive correlation between 1-hydroxypyrene (1-OHP), a biomarker for PAH exposure from COFs, and 8-Hydroxy-2′-Deoxyguanosine (8-OHdG), a marker of oxidative stress [27]. This positive correlation between 1-OHP and 8-OHdG has also been observed in Chinese restaurant workers [28].

According to Glazer et al (Glazer et al, 1985 AJR), the short-axis diameters for 4R and 4L lymph nodes were 3.2 ± 2.0 mm and 2.1 ± 1.6 mm, respectively. These measurements are closer to the lymph node size observed in the control group of our study (4R/10R, 3.13 ± 1.36 mm; 4L/10L, 1.98 ± 1.27 mm), while the lymph nodes in school cooks were slightly larger (4R/10R, 4.12 ± 1.66 mm; 4L/10L, 2.63 ± 1.50 mm). The normal lymph node short-axis diameter values further demonstrate that the school cooks’ lymph nodes are larger than those of the general population. The lymph nodes serve as the first line of defense against external pathogens, and lymph node disorders have been reported to be associated with various chemical exposures [29]. In cases of high dust exposure, such as in coal workers, dust deposition within the lymph nodes has been observed [30]. Cigarette dust accumulation in smokers is known to cause enlargement of the hilar and mediastinal lymph nodes [31]. Based on this, we hypothesized that the accumulation of COFs could also lead to lymph node enlargement. In this study, significant size differences in certain lymph nodes were observed among the school cooks group, suggesting a meaningful immunological response to external substances. Radiologically, such findings are noteworthy for clinicians as increased attention has been directed toward lung cancer screening in school cooks, with the widespread use of low-dose CT for early detection [32]. In school cooks, lymph node enlargement is frequently observed without other pathological lung imaging findings, such as prominent pneumoconiosis, which may influence clinical decision-making processes in diagnosing and treating patients with suspected malignancies. Since lymph node enlargement in malignancy is often considered a sign of metastatic disease, the lymph node enlargement observed in Cooks exposed to COFs could complicate preoperative staging because CT relies heavily on size for distinguishing between benign and malignant lymph nodes. This highlights the need for careful consideration when evaluating lymph node involvement in cancer staging in individuals exposed to COFs.

Furthermore, our findings revealed central bronchial wall thickening in school cooks but not at small airways. This aligns with prior research showing that the inflammation and airway remodeling associated with chronic bronchitis predominantly occur in the central airways [33]. In previous studies, PM_2.5_ concentrations measured in Korean school cafeteria cooking facilities had a maximum value of 367.90 μg/m³, while PM_10_ levels showed a maximum value of 6169.00 μg/m³ [2]. For particles in the 1–10 μm range, the tracheobronchial region generally has a lower deposition rate than the alveolar region, though particles around 5 μm in diameter have a relatively high deposition potential [34]. Conversely, ultrafine particles below 0.1 μm may exhibit higher deposition rates in the tracheobronchial region than in the alveolar region [35].

In domestic cooking environments in the Republic of Korea, frying pork has been reported to generate a large number of particles around 100–180 nm [36]. Furthermore, studies on particles from various cooking oils have shown that ultrafine particles between 10 and 100 nm constitute a substantial proportion of PM_2.5_, with a reported fraction as high as 0.76–0.99 [37]. Therefore, COFs may contain a considerable number of particles approximately 5 μm or <0.1 μm in size, likely to deposit in the large airways, potentially causing significant inflammation and leading to bronchial wall thickening.

In our study, the prevalence of solid nodules was higher among cooks than in the control group. The prevalence of pulmonary nodules in the general population varies, with one study reporting a prevalence of 33% and a meta-analysis estimating around 30% (Callister ME et al Thorax 2015, D Chen et al JTD 2024). In our study, the control group had a similar prevalence of 33%, while school cooks had a notably higher prevalence of 75%. Positive screening results corresponding to Lung-RADS were observed in 11.4% of school cooks and 2.3% of controls. These findings reflect a slightly higher rate than another study on Korean cafeteria workers, which reported an 8.4% positivity rate [32]. A limitation of previous studies was the significant age difference between the two groups. In this study, we addressed this limitation by creating a nonexposed control group matched by age. Nevertheless, the results of this study (2.3%) were at a similar level to the positivity rate in the control group (2.8%). In a retrospective cohort study of 4,365 never-smoking women aged 40 to 74 years in the Republic of Korea, the initial low-dose CT positivity rate was 6.7% [38]. Similarly, another cohort study involving 12,176 never-smokers found positive results in 2.1% of participants [39]. Conversely, in the Korean Lung Cancer Screening Project (K-LUCAS), which included current smokers aged 54 years and older with a smoking history of at least 30 pack-years, positive screening results were found in 1,868 out of 11,394 participants (16.0%) [40]. In summary, the positive screening rate among school cooks was higher than that of nonexposure controls and never-smoking women but lower than the rate observed in smokers.

This study has a significant limitation in that it was conducted retrospectively, thereby restricting a comprehensive analysis of various confounders that could potentially influence the results, such as prior occupational history, social economic status, environmental exposure to respiratory toxicants, and pre-existing respiratory conditions aside from age and sex. In particular, the absence of data on smoking history and detailed exposure information is a notable shortcoming. However, the smoking prevalence among women in the Republic of Korea is relatively low, with a self-reported rate of 6.8% [41]. Additionally, a previous study conducted on school cooking staff in the Republic of Korea reported that all 167 cooks and 36 control participants responded as nonsmokers in the survey, with no significant difference in passive smoking exposure between the groups [42]. Based on these findings, it is not considered plausible that the cooking staff group exhibited a disproportionately high smoking prevalence. A notable limitation of this study is the inability to conduct stratified analyses based on detailed occupational history (such as school type, daily exposure duration, cooking volume, and total exposure period) due to the retrospective nature of the data. Nevertheless, it is relatively clear that the cooking staff group included in the study had a minimum of 10 years of exposure, which enhances the comparability of the selected participants. Currently, the use of low-dose CT scans among school cooks in the Republic of Korea is increasing, highlighting the need for expanded research on the health effects on Korean school cooks through comprehensive assessments of occupational history and work environment.

This study has several other limitations. This study utilized a retrospective, cross-sectional analysis of medical records and imaging data, which inherently limits the ability to infer temporal relationships or establish causality. Although the study presents various statistical metrics and consistently demonstrates significant radiological changes in the cooking staff group, it is important to note that these changes are subclinical in nature. Therefore, caution should be exercised when interpreting these findings as they do not directly imply an increased risk of clinical symptoms or other diseases, such as cancer. Future research using a longitudinal design would be necessary to better understand the clinical relevance and potential long-term risks associated with these radiological changes. Second, measurements of bronchial wall thickening and lymph node diameter were conducted manually by an experienced radiologist, rather than using specific software. To ensure accuracy, a certified thoracic radiologist independently reviewed the CT images, confirmed the results, and reached a consensus with the initial radiologist.

In cooking environments, various harmful substances are present, yet the nature of the exposure can vary significantly due to differences in cultural and dietary practices. Particularly in group facilities or restaurants, exposure to hazardous substances can be high in concentration, prolonged in duration, and extended over long periods. However, there are limitations in applying foreign research findings, as dietary habits and cooking cultures differ, emphasizing the need for independent studies on health impacts within specific contexts.

This study utilized low-dose CT screening data from school cooks to examine various radiological indicators. The findings suggest that long-term employment as a school cooks may lead to subclinical health effects on the body. For rare but serious diseases like lung cancer, large-scale, long-term studies are necessary to reveal any significant differences in prevalence. However, even before results on the prevalence of these serious but low-frequency diseases are available, industrial hygiene and public health management measures are warranted. The findings from this study can serve as evidence to support the allocation of resources for research and public health measures aimed at safeguarding the health of school cooks.

## CRediT authorship contribution statement

**Jung Hee Hong:** Writing – original draft, Visualization, Software, Investigation. **Jin Young Kim:** Writing – review & editing, Validation. **Kiook Baek:** Writing – review & editing, Supervision, Methodology, Data curation, Conceptualization.

## Declaration of Generative AI and AI-assisted technologies in the writing process

During the preparation of this work, the authors used ChatGPT for English translation and polishing. After using this tool/service, the authors reviewed and edited the content as needed and take full responsibility for the content of the publication.

## Funding

No funding was obtained for this work.

## Conflicts of interest

The authors declare that they have no known competing financial interests or personal relationships that could have appeared to influence the work reported in this paper.
